# A survey of physician receptivity to molecular diagnostic testing and readiness to act on results for early-stage colon cancer patients

**DOI:** 10.1186/s12885-016-2812-1

**Published:** 2016-10-03

**Authors:** Ronald E. Myers, Thomas Wolf, Phillip Shwae, Sarah Hegarty, Stephen C. Peiper, Scott A. Waldman

**Affiliations:** 1 Department of Medical Oncology, Thomas Jefferson University, Benjamin Franklin House, Suite 314, 834 Chestnut St, Philadelphia, PA 19107 USA; 2 Thomas Jefferson University, 305 South 11th Street, Apt. 4F, Philadelphia, PA 19107 USA; 3 Department of Pharmacology & Experimental Therapeutics, Thomas Jefferson University, 1015 Chestnut Street Building, Suite M-100 Mezzanine, 1015 Chestnut Street, Philadelphia, PA 19107 USA; 4 Department of Pathology, Anatomy and Cell Biology, Thomas Jefferson University, Jeff Hall, Room 279, 1020 Locust St, Philadelphia, PA 19107 USA

**Keywords:** Decision analysis, Cancer, Colon carcinogenesis, Molecular genetics, Staging

## Abstract

**Background:**

We sought to assess physician interest in molecular prognosic testing for patients with early stage colon cancer, and identify factors associated with the likelihood of test adoption.

**Methods:**

We identified physicians who care for patients with early-stage (pN0) colon cancer patients, mailed them a survey, and analyzed survey responses to assess clinician receptivity to the use of a new molecular test (GUCY2C) that identifies patients at risk for recurrence, and clinician readiness to act on abnormal test results.

**Results:**

Of 104 eligible potential respondents, 41 completed and returned the survey. Among responding physicians, 56 % were receptive to using the new prognostic test. Multivariable analyses showed that physicians in academic medical centers were significantly more receptive to molecular test use than those in non-academic settings.

Forty-one percent of respondents were ready to act on abnormal molecular test results. Physicians who viewed current staging methods as inaccurate and were confident in their capacity to incorporate molecular testing in practice were more likely to say they would act on abnormal test results.

**Conclusions:**

Physician receptivity to molecular diagnostic testing for early-stage colon cancer patients is likely to be influenced by practice setting and perceptions related to delivering quality care to patients.

**Trial registration:**

ClinicalTrials.gov Identifier: NCT01972737

## Background

Advances in identifying novel markers and related clinical targets, along with the emergence of new diagnostic techniques and the development of pharmacologic antagonists of key signaling elements have generated expectations of dramatic change in the care of patients diagnosed with cancer. New and emerging molecular diagnostic tests have the potential to improve the accuracy of disease staging, determine if a given patient may be predisposed to disease progression, and provide useful information about the patient’s likely response to treatment.

In the age of personalized medicine, patients are becoming increasingly aware of and are asking physicians about the value of such testing. Physicians who care for cancer patients are challenged by the need to learn about new developments in the field and the demand to apply these new tools in patient care [1]. Realizing the potential benefits of molecular diagnostic testing in cancer care will require high levels of physician receptivity and readiness to use such tests routinely [2, 3]. To date, however, limited research has reported on physician receptivity to and use of molecular diagnostic testing in cancer care [4].

In a recent survey, 75 % of physicians who treat cancer patients said they believe the use of genomic testing can improve patient care. However, respondents also stated that they had ordered genomic testing for only 4 % of their patients [5]. In another study, 90 % of respondents reported that they supported genomic testing, but less than half felt confident in their ability to interpret the test results to their patients [6]. Similar findings were noted by Stanek et al. [7] who found that 98 % of physicians surveyed viewed molecular risk assessment as having the potential to play a crucial role in determining drug therapies for patients, but only 10 % felt confident in their understanding to best use test results to guide the process of prescribing treatment.

The study reported here focuses on guanylate cyclase C (GUCY2C) testing, a new molecular diagnostic test that has been developed for use in conjunction with histopathology to guide treatment decision making for patients with early-stage (pN0) colon cancer. Conventional histopathological analysis for such patients is routinely triggered at the time of diagnosis (“reflex tests”), and therapy is to a large degree based on the pathologic stage that is determined. GUCY2C is a protein expressed normally by intestinal epithelial cells, but is universally over-expressed by metastatic colon tumors [8–10]. There is a strong association between the expression of GUCY2C in regional lymph nodes, measured using a validated quantitative RT-PCR assay [11], and the development of recurrent metastatic disease in otherwise lymph node-negative patients [12, 13]. Moreover, this test has been externally validated and commercialized [14–17].

The current investigation was part of a larger clinical trial (NCT01972737), which focused on the utility of GUCY2C as a vaccine target for the secondary prevention of metastatic colorectal cancer. Here, we collected and analyzed survey data from clinicians in the Greater Philadelphia Area who treat colon cancer patients. Main objectives were to: (1) assess physician receptivity to GUCY2C testing, (2) assess physician readiness to act on test results, and (3) identify factors associated with test receptivity and readiness to act.

## Methods

The research team initially obtained from the Department of Strategy and Business Development at Thomas Jefferson University a mailing list of medical oncologists, surgeons, and gastroenterologists practicing in the Greater Philadelphia Area. Following established methods [18], we sent all physicians on the mailing list an introductory letter that described the purpose of the study and invited response via provision of written consent. In addition, we requested that recipients of the invitation complete and return an enclosed survey questionnaire using a postage-paid return envelope or to complete an online version of the survey. The mailing also advised the recipient that s/he would be compensated ($125) for completion of the survey. We also included a postcard in the mailing that allowed the recipient to opt-out of the study. A month after this initial mailing, the research team sent non-respondents another study invitation, which included a copy of the survey questionnaire, an opt-out card, and a return envelope. At 60 days, the research team attempted to contact non-respondents by telephone to encourage response.

At the beginning of the survey, GUCY2C and testing were described as follows: “GUCY2C is a protein expressed normally by intestinal epithelial cells, but is universally over-expressed by metastatic colon tumors. There is a relationship between the categorical (yes/no) presence of occult tumor cells in lymph nodes detected by GUCY2C testing and prognosis in pN0 colon cancer. This paradigm has been used to quantify occult tumor burden in nodes. The GUCY2C test could be ordered for colon cancer patients at the time of surgery. Results of this test could be used in conjunction with histopathology to inform the clinical decision to or not to recommend chemotherapy.” Survey items that operationalized constructs drawn from an explanatory framework known as the Diagnostic Evaluation Model (DEM) [19] followed this scenario.

DEM items measured factors that could help to explain physician receptivity to GUCY2C testing and readiness to act on test results. These factors include physician practice environment, sociodemographic background characteristics and experience, perceptions about testing, and perceptions about treatment. Regarding practice environment, respondents were asked whether they practiced mainly in an academic medical center, community-based hospital, and other practice settings. We asked respondents to provide information on background characteristics (i.e., age, gender, race, ethnicity, and years in practice) and experience caring for colon cancer patients (i.e., exposure to pN0 colon cancer patients and patients who experienced recurrence).

To elicit perceptions about testing, we asked respondents to report how accurate they thought histopathology, GUCY2C testing, and combined histopathology and GUCY2C testing are in staging pN0 colon cancer patients (i.e., not accurate, somewhat accurate, very accurate). In addition, we asked whether physicians agreed (Strongly Disagree – 1 versus Strongly Agree - 5) that each of these three approaches to testing could provide sufficient information that was needed to recommend treatment. An item that assessed respondent agreement with the view that current testing methods were sufficiently accurate for pN0 colon cancer patients was also included.

Respondent perceptions about treatment were measured by assessing physician stress from uncertainty using five items from the Physicians’ Reactions to Uncertainty (PRU) scale (α = 0.59) [20]. Single items were used to measure physician perceived ease of making a treatment decision for pN0 colon cancer patients, confidence in identifying an effective treatment for early-stage colon cancer patients, and confidence that molecular diagnostic testing could improve patient treatment.

We assessed physician interest in the new test along two dimensions, receptivity and readiness to act. Specifically, we determined physician receptivity to the test by eliciting level of agreement (Strongly Disagree – 1 to Strongly Agree - 5) with the following statements: “I think GUCY2C test results should be considered when treatment is recommended for pN0 colon cancer patients.” and “I think all patients with pN0 colon cancer should have a GUCY2C test.” The summed mean responses to these items were dichotomized as <3 (disagree or neutral) versus >3 (agree) to determine clinician receptivity to the use of GUC2YC testing. Survey respondents were determined to be receptive to testing if the mean response to the two survey items was >3. In terms of readiness to act, we asked physicians to respond to the following statement: “I would treat patients with pN0 colon cancer who have abnormal GUCY2C test results more aggressively than patients with a normal test result.” Responses were dichotomized as ≤3 (disagree or neutral) versus >3 (agree)” to measure clinician readiness to act on abnormal test results. Physicians were considered to be ready to act on abnormal test results if their response to this single item was >3. Finally, we included open-ended questions that allowed respondents to report factors that would influence them to order GUCY2C testing.

Fisher’s Exact testing was used to assess statistically significant associations between categorical variables and the two outcomes, while the Wilcoxon test was used to assess associations of continuous variables with outcomes. Covariates associated with the outcome variables at the *p* ≤ 0.2 level were included in a multivariable logistic regression models. Backwards selection was used to determine the model, with retention of those independent variables that were associated at *p*-value of 0.05. Because of the small sample size, exact *p*-values are reported.

Members of the research team (RM, TW, and PS) reviewed comments reported by physicians on the factors that would influence them to and not to order testing, and generated a set of unique factor categories. Independently, TW and PS assigned each factor to a category and then resolved any discrepancies in joint consultation with RM. Category frequencies were generated.

## Results

A total of 211 physicians were targeted to receive the mailed survey. Feedback from that initial mailing, a subsequent reminder mailing, and a final telephone reminder resulted in the exclusion of 60 physicians with incomplete contact information. Additionally, 47 individuals were excluded, because they reported that they did not currently see pN0 colon cancer patients, and two physicians were found to be deceased. Thus, there were 104 physicians who were eligible and available to complete the mailed survey. Of this number, 43 (41 %) completed the survey, 18 (17 %) declined to participate, and 43 (41 %) were lost to follow-up. The research team decided to remove two gastroenterologists from the pool of respondents because it was determined that physicians in this specialty are unlikely to recommend molecular testing for cancer patients following initial diagnosis. Thus, 41 respondents were included in the final analyses.

Survey DEM measures are displayed in Table 1. Study outcomes displayed in Table 1 show that overall, 51 % of respondents were <50 years of age; 83 % were male; and 68 % were white. Forty-nine percent of physicians agreed that GUCY2C test results should be used to guide treatment recommendations for patients with early-stage colon cancer, and 44 % percent agreed that all early-stage colon cancer patients should have such testing. A total of 18 respondents had a two-item summed mean response ≤3, and 22 respondents had a summed receptivity score that was >3. The respective percentage of respondents in these categories, which are not included in the table, are 44 and 56 %, respectively. The numbers of respondents in these categories corresponds to the table column headings for the outcomes.

**Table 1:** Univariable associations of physician receptivity to genomic risk assessment (GUCY2C) for pN0 colon cancer patients

	Total (*n* = 41)	Receptive (*n* = 22)	Not Receptive (*n* = 19)	*p*-value
	n (%)	n (%)	n (%)	
Practice Environment:				
Practice Setting				0.004
Community-based or Other	24 (58.54)	8 (33.33)	16 (66.67)	.
Academic Center-based	17 (41.46)	14 (82.35)	3 (17.65)	.
Sociodemographic Background and Experience:				
Age				1.000
<50	21 (51.22)	11 (52.38)	10 (47.62)	.
50+	20 (48.78)	11 (55.00)	9 (45.00)	.
Gender				0.219
Male	34 (82.93)	20 (58.82)	14 (41.18)	.
Female	7 (17.07)	2 (28.57)	5 (71.43)	.
Race				1.000
(missing/unknown)	1 (.)	0 (.)	1 (.)	.
Asian	13 (32.50)	7 (53.85)	6 (46.15)	.
White	27 (67.50)	15 (55.56)	12 (44.44)	.
Hispanic/Latino				0.463
No	40 (97.56)	22 (55.00)	18 (45.00)	.
Yes	1 (2.44)		1 (100.0)	.
Years in Practice				0.891
< =10	4 (9.76)	2 (50.00)	2 (50.00)	.
10–20	11 (26.83)	5 (45.45)	6 (54.55)	.
>20	26 (63.41)	15 (57.69)	11 (42.31)	.
Specialty				0.758
Medical Oncologists+	23 (56.10)	13 (56.52)	10 (43.48)	.
GI Surgeons	18 (43.90)	9 (50.00)	9 (50.00)	.
Number of newly diagnosed pN0 colon cancer patients seen in past 12 months				0.668
<5	6 (14.63)	4 (66.67)	2 (33.33)	.
5+	35 (85.37)	18 (51.43)	17 (48.57)	.
Percentage of pN0 colon cancer patients who have had a recurrence in the past 12 months				1.000
None	20 (48.78)	11 (55.00)	9 (45.00)	.
1+	21 (51.22)	11 (52.38)	10 (47.62)	.
On average how many pN0 colon cancer patients do you see each month?				0.737
<5	28 (68.29)	16 (57.14)	12 (42.86)	.
5+	13 (31.71)	6 (46.15)	7 (53.85)	.
Perceptions about Diagnostic/Prognostic Testing:				
Belief in accuracy of histopathology				1.000
Not Agree	14 (34.15)	8 (57.14)	6 (42.86)	.
Agree	27 (65.85)	14 (51.85)	13 (48.15)	.
Belief in accuracy of GUY2C testing				0.216
Not Agree	17 (41.46)	7 (41.18)	10 (58.82)	.
Agree	24 (58.54)	15 (62.50)	9 (37.50)	.
Belief in accuracy of combined histopathology and GUCY2C testing				0.038
Not Agree	4 (9.76)	0 (0.0)	4 (100.0)	.
Agree	37 (90.24)	22 (59.46)	15 (40.54)	.
Belief in need for a more accurate prognostic test for pN0 colon cancer patients				1.000
Not Agree	2 (4.88)	1 (50.00)	1 (50.00)	.
Agree	39 (95.12)	21 (53.85)	18 (46.15)	.
Belief that current staging methods are not accurate for pN0 colon cancer patients				0.216
Not Agree	17 (41.46)	7 (41.18)	10 (58.82)	.
Agree	24 (58.54)	15 (62.50)	9 (37.50)	.
Accuracy of histopathology alone				0.388
(missing/unknown)	1 (.)	0 (.)	1 (.)	.
Very Accurate	6 (15.00)	3 (50.00)	3 (50.00)	.
Somewhat Accurate	31 (77.50)	16 (51.61)	15 (48.39)	.
Not Accurate	3 (7.50)	3 (100.0)	0 (0.0)	.
Don’t know	0 (0.0)	0 (0.0)	0 (0.0)	.
Accuracy of GUCY2C testing alone				0.133
(missing/unknown)	2 (.)	0 (.)	2 (.)	.
Very Accurate	6 (15.38)	4 (66.67)	2 (33.33)	.
Somewhat Accurate	22 (56.41)	13 (59.09)	9 (40.91)	.
Not Accurate	3 (7.69)	3 (100.0)	0 (0.0)	.
Don’t Know	8 (20.51)	2 (25.00)	6 (75.00)	.
Accuracy of combined histopathology and GUCY2C testing				0.054
(missing/unknown)	1 (.)	0 (.)	1 (.)	.
Very Accurate	25 (62.50)	16 (64.00)	9 (36.00)	.
Somewhat Accurate	11 (27.50)	6 (54.55)	5 (45.45)	.
Not Accurate	0 (0.0)	0 (0.0)	0 (0.0)	
Don’t Know	4 (10.00)	0 (0.0)	4 (100.0)	.
Perceptions about Treatment:				
Stress from uncertainty in decision making about treatment for pN0 colon cancer patients (scale)				0.350
Not Agree (<=3)	22 (53.66)	10 (45.45)	12 (54.55)	.
Agree (>3)	19 (46.34)	12 (63.16)	7 (36.84)	.
Belief that treatment decision making for pN0 colon cancer patients is easy				0.009
Not Agree	13 (31.71)	11 (84.62)	2 (15.38)	.
Agree	28 (68.29)	11 (39.29)	17 (60.71)	.
Confidence in treatment decision making for pN0 colon cancer patients				0.325
Not Agree	12 (29.27)	8 (66.67)	4 (33.33)	.
Agree	29 (70.73)	14 (48.28)	15 (51.72)	.
Belief that treatment choice for pN0 colon cancer patients is clear				0.756
Not Agree	19 (46.34)	11 (57.89)	8 (42.11)	.
Agree	22 (53.66)	11 (50.00)	11 (50.00)	.

In univariable analyses (Table 1), the following variables were associated with physician receptivity to GUCY2C testing: practice located in an academic medical center (*p* = 0.004); belief that the combined results of histopathology and GUCY2C testing could provide information needed to recommend treatment (*p* = 0.038); belief that GUCY2C testing alone, as well as combined histopathology and GUCY2C testing were somewhat or very accurate (*p* = 0.133 and *p* = 0.054, respectively); and belief that making treatment decisions for pN0 colon cancer patients is easy (*p* = 0.009). Multivariable analysis results (Table 2) indicate that physicians who practiced in academic medical centers were more receptive to GUCY2C testing than those who practiced in community hospitals or other settings (OR = 7.14, CI: 1.28, 55.02).

**Table 2:** Multivariable logistic regression on combined D1, D3 outcome (D1, D3 mean >3) (*N* = 41)

Exact odds ratios				
Parameter	Estimate	95 % Confidence limits		Two-sided *p*-value
How would you describe your practice setting?				
Hospital-based vs. Community Based/Other	7.14	1.28	55.02	0.021

In terms of readiness to act (Table 3), there were 24 physicians had a response <3, while 17 clinicians who had a response to the one item used to assess readiness that was >3. The respective percentages of respondents in these categories, which are not displayed in the table, are 59 and 41 %, respectively. Univariable analyses showed the following variables to be associated with physician readiness to act on abnormal test results: race (*p* = 0.103); belief that GUCY2C testing alone and the combined results of histopathology and GUCY2C testing could provide information needed to recommend treatment (*p* = 0.012 and *p* = 0.128, respectively); belief that current staging methods are not accurate for pN0 colon cancer patients (0.062); belief that GUCY2C testing alone, as well as the combined results of histopathology and GUCY2C testing are somewhat or very accurate (*p* = 0.179 and *p* = 0.084, respectively); belief that treatment choice is clear (*p* = 0.025).

**Table 3:** Univariable associations of physician readiness to act on genomic risk assessment (GUCY2C) for pN0 colon cancer Patients

Variable	Total (*n* = 41)	Ready (*n* = 17)	Not ready (*n* = 24)	*p*-value
	n (%)	n (%)	n (%)	
Practice Environment:				
Practice Setting				0.748
Community-based or Other	24 (58.54)	9 (37.50)	15 (62.50)	.
Academic Center-based	17 (41.46)	8 (47.06)	9 (52.94)	.
Sociodemographic Background and Experience:				
Age				0.350
<50	21 (51.22)	7 (33.33)	14 (66.67)	.
50+	20 (48.78)	10 (50.00)	10 (50.00)	.
Gender				1.000
Male	34 (82.93)	14 (41.18)	20 (58.82)	.
Female	7 (17.07)	3 (42.86)	4 (57.14)	.
Race				0.103
(missing/unknown)	1 (.)		1 (.)	.
Asian	13 (32.50)	3 (23.08)	10 (76.92)	.
White	27 (67.50)	14 (51.85)	13 (48.15)	.
Hispanic/Latino				1.000
No	40 (97.56)	17 (42.50)	23 (57.50)	.
Yes	1 (2.44)		1 (100.0)	.
Years in Practice				0.559
< =10	4 (9.76)	2 (50.00)	2 (50.00)	.
10–20	11 (26.83)	3 (27.27)	8 (72.73)	.
>20	26 (63.41)	12 (46.15)	14 (53.85)	.
Specialty				1.000
Medical Oncologists+	23 (56.10)	10 (43.48)	13 (56.52)	.
GI Surgeons	18 (43.90)	7 (38.89)	11 (61.11)	.
Number of newly diagnosed pN0 colon cancer patients seen in past 12 months				0.679
<5	6 (14.63)	3 (50.00)	3 (50.00)	.
5+	35 (85.37)	14 (40.00)	21 (60.00)	.
Percentage of pN0 colon cancer patients who have had a recurrence in the past 12 months				0.530
None	20 (48.78)	7 (35.00)	13 (65.00)	.
1+	21 (51.22)	10 (47.62)	11 (52.38)	.
On average how many pN0 colon cancer patients do you see each month?				0.499
<5	28 (68.29)	13 (46.43)	15 (53.57)	.
5+	13 (31.71)	4 (30.77)	9 (69.23)	.
Perceptions about Diagnostic/Prognostic Testing				
Belief in accuracy of histopathology				0.742
Not Agree	14 (34.15)	5 (35.71)	9 (64.29)	.
Agree	27 (65.85)	12 (44.44)	15 (55.56)	.
Belief in accuracy of GUY2C testing				0.012
Not Agree	17 (41.46)	3 (17.65)	14 (82.35)	.
Agree	24 (58.54)	14 (58.33)	10 (41.67)	.
Belief in accuracy of combined histopathology and GUCY2C testing				0.128
Not Agree	4 (9.76)		4 (100.0)	.
Agree	37 (90.24)	17 (45.95)	20 (54.05)	.
Belief in need for a more accurate prognostic test for pN0 colon cancer patients				1.000
Not Agree	2 (4.88)	1 (50.00)	1 (50.00)	.
Agree	39 (95.12)	16 (41.03)	23 (58.97)	.
Belief that current staging methods are not accurate for pN0 colon cancer patients				0.062
Not Agree	17 (41.46)	4 (23.53)	13 (76.47)	.
Agree	24 (58.54)	13 (54.17)	11 (45.83)	.
Accuracy of histopathology alone				0.861
(missing/unknown)	1 (.)	0 (.)	1 (.)	.
Very Accurate	6 (15.00)	2 (33.33)	4 (66.67)	.
Somewhat Accurate	31 (77.50)	14 (45.16)	17 (54.84)	.
Not Accurate	3 (7.50)	1 (33.33)	2 (66.67)	.
Don’t Know	0 (0.0)	0 (0.0)	0 (0.0)	
Accuracy of GUCY2C testing alone				0.179
(missing/unknown)	2 (.)	0 (.)	2 (.)	.
Very Accurate	6 (15.38)	5 (83.33)	1 (16.67)	.
Somewhat Accurate	22 (56.41)	9 (40.91)	13 (59.09)	.
Not Accurate	3 (7.69)	1 (33.33)	2 (66.67)	.
Don’t Know	8 (20.51)	2 (25.00)	6 (75.00)	.
Accuracy of combined histopathology and GUCY2C testing				0.084
(missing/unknown)	1 (.)	0 (.)	1 (.)	.
Very Accurate	25 (62.50)	14 (56.00)	11 (44.00)	.
Somewhat Accurate	11 (27.50)	2 (18.18)	9 (81.82)	.
Not Accurate	0 (0.0)	0 (0.0)	0 (0.0)	
Don’t Know	4 (10.00)	1 (25.00)	3 (75.00)	.
Perceptions about Treatment				
Stress from uncertainty in decision making about treatment for pN0 colon cancer patients (scale)				0.216
Not Agree (<=3)	22 (53.66)	7 (31.82)	15 (68.18)	.
Agree (>3)	19 (46.34)	10 (52.63)	9 (47.37)	.
Belief that treatment decision making for pN0 colon cancer patients is easy				0.499
Not Agree	13 (31.71)	4 (30.77)	9 (69.23)	.
Agree	28 (68.29)	13 (46.43)	15 (53.57)	.
Confidence in treatment decision making for pN0 colon cancer patients				0.729
Not Agree	12 (29.27)	4 (33.33)	8 (66.67)	.
Agree	29 (70.73)	13 (44.83)	16 (55.17)	.
Belief that treatment choice for pN0 colon cancer patients is clear				0.025
Not Agree	19 (46.34)	4 (21.05)	15 (78.95)	.
Agree	22 (53.66)	13 (59.09)	9 (40.91)	.

Multivariable analysis results (Table 4) indicate that physicians respondents who thought that current staging methods for pN0 colon patients were not sufficiently accurate were more likely to report that they would act on abnormal GUCY2C test results than those who did not hold this belief (OR = 5.98, CI: 1.05, 49.32). In addition, physicians who stated that it was clear what treatment choice is correct for their patients were significantly more likely to say they would act on GUY2C test results than those who were less confident (OR = 7.74, CI: 1.41, 62.20).

**Table 4:** Multivariable logistic regression of physician readiness to act on genomic risk assessment (GUCY2C) for pN0 colon cancer patients (*N* = 41)

Exact odds ratios				
Parameter	Estimate	95 % Confidence limits		Two-sided *p*-value
It is clear what treatment choice is right for my patients.				
Agree vs. Do Not Agree	7.74	1.41	62.20	0.013
I am not satisfied with the accuracy of current approaches for staging my patients.				
Agree vs. Do Not Agree	5.98	1.05	49.32	0.042

All 41 physicians responded to the open-ended survey question that asked them to indicate the factors that would discourage them from recommending GUCY2C testing. Together, the respondents identified a total of 85 factors. Of this number, 31 (36 %) factors related to having insufficient information about test accuracy; 26 (31 %) expressed concern about the cost of such testing; 22 (26 %) said they were were not familiar with guidelines that specified circumstances for test use; and 9 (11 %) reported that they did not think the test was readily available.

## Discussion

This study is the first to measure physician interest in molecular testing in terms of receptivity and readiness to act on test results. In addition, the study is novel in that it focuses attention on the testing for early-stage colon cancer patients. Moreover, the study used an explanatory framework (DEM) to identify factors that influence physician receptivity to test use and readiness to act on test results.

We found that 44 % of surveyed physicians were receptive to GUCY2C testing for patients with early-stage colon cancer. To our knowledge, this report is the first instance in which physician receptivity to prognostic molecular diagnostic testing in this patient population been assessed. Elsewhere, it has been reported that more than half of physicians who treat metastatic colorectal cancer patients ordered a genetic test for their patients after it was cited in clinical guidelines [2]. This level of interest in molecular diagnostic testing suggests that many physicians who treat early-stage patients see a need to improve on existing staging methods, and have a desire to consider test results in treatment planning.

We also determined that physicians who practiced at academic medical centers were more enthusiastic about molecular testing for risk of recurrence than their counterparts in community hospital settings. This finding may reflect the fact that molecular diagnostic testing is more readily available in academic medical centers than in community settings, as such testing can be provided in-house. As a result, tests in academic settings can be performed easily and their results obtained in a timely manner to inform physician recommendations. It may also be the case that there may be more opportunities for physicians in academic medical centers to share information about new molecular diagnostic tests, personal experiences using the tests, and patient outcomes. As a result, academic medical center-based physicians may feel a greater sense confidence in using the tests in routine care.

Forty-one percent of physicians in the current study reported that they were ready to treat patients with an abnormal GUYC2C test result more aggressively than patients who had a normal test result. We found that physicians who were ready to act on GUCY2C test results were more likely to question the accuracy of current staging methods than physicians who were not ready to act of test results. Notably, 90 % of physicians reported that the combined histopathology and molecular diagnostic testing could provide sufficient information to allow them to make good treatment decisions. Most physicians tended to view molecular diagnostic testing as providing them with additional information that, in conjunction with current histopathology results, could help to better-forecast recurrence, and improve their capacity to recommend the most appropriate treatment. In the context of the current investigation, we found that respondents who reported being ready to treat patients with abnormal GUCY2C results more aggressively were more likely to believe that molecular testing could improve their capacity to provide high quality care to their patients.

Physician readiness to act on abnormal test results may be influenced to some extent by practitioner feelings of stress from uncertainty about treating early-stage colon cancer patients on the basis of histopathology analyses alone, and confidence in interpreting molecular test results. In other reports, physicians who have limited familiarity with molecular diagnostic testing have expressed uncertainty about their capacity to interpret and explain test results to their patients, and, as a consequence, may be more reluctant to support the use of such tests in practice [4, 6, 7].

It is important to mention that the generalizability of findings reported here might be limited for several reasons. First, the survey response rate among eligible physicians was less than 50 %. Therefore, survey responses may not be representative of physicians who care for patients diagnosed with early-stage colon cancer. In addition, the survey was administered to physicians in one geographic area; and, perceptions related to molecular diagnostic testing may reflect those held by practitioners in this region. In addition, the number of physicians who completed the survey is small, thus, findings from our analyses may be spurious. It is also the case that we collected data on only physician perceptions and receptivity to the use of only one molecular test for a specified set of cancer patients. Findings may vary if another type of test had been presented and a different set of patients were referenced. Social desirability in survey response may be another challenge to generalizabilty, as respondents could have been motivated to provide what they may viewed as a “correct” response to survey items.

## Conclusion

Health care institutions and groups committed to developing state-of-the-art practice guidelines are placing greater emphasis on physician education about the use of laboratory-developed molecular tests in personalized medicine [21]. Research on a national cohort of clinicians is needed to identify factors that influence physician uptake of molecular testing for cancer patients and to determine the impact of test results on clinical recommendations that are made. Furthermore, research should also focus attention on assessing patient outcomes that result from using these new evidence-based diagnostic and prognostic methods.

In the future, there will be a steady increase in predictive molecular testing in cancer care. The adoption of such testing will be influenced by a variety of factors, including organizational factors and provider characteristics and perceptions. Teng et al. have concluded that evidence suggests that the adoption of molecular testing in routine care is most likely to take place when this service is performed as a laboratory test commonly performed at the time of diagnosis, rather than requiring a discretionary order for testing, and when payers routinely reimburse testing [22]. It is expected that ongoing efforts to include molecular diagnostic testing in clinical guidelines will add momentum to this process [23]. Findings reported in the current study highlight the need to address physician educational and decision support needs to advance the use of molecular testing.

Furthermore, the movement towards patient-centered care highlights the importance of physician-patient shared decision-making about diagnostic testing and treatment. Decision support interventions that are designed to provide patients with information, elicit values and preferences related to treatment options, and facilitate shared decision making must also be integrated into clinical practice. Centers of excellence in medical care must lead the way by deploying effective methods for overcoming structural obstacles, operational barriers, and individual limitations to molecular test uptake. This effort should include support for shared decision making in health systems and the prospective assessment of clinical outcomes.

## Abbreviations

DEM: Diagnostic evaluation model
GUCY2C: Guanylate cyclase C
IRB: Institutional review board
pN0: No regional lymph node metastasis identified histologically
PRU: Physicians’ reactions to uncertainty
